# *Lactobacillus amylovorus* KU4 ameliorates diet-induced obesity in mice by promoting adipose browning through PPARγ signaling

**DOI:** 10.1038/s41598-019-56817-w

**Published:** 2019-12-27

**Authors:** Sung-Soo Park, Yeon-Joo Lee, Hyuno Kang, Garam Yang, Eun Jeong Hong, Jin Yeong Lim, Sejong Oh, Eungseok Kim

**Affiliations:** 1 Department of Biological Sciences, College of Natural Sciences, Chonnam National University, 77 Yongbong-ro, Buk-gu, Gwangju 61186 Republic of Korea; 2 Division of Animal Science, College of Agriculture & Life Science, Chonnam National University, 77 Yongbong-ro, Buk-gu, Gwangju 61186 Republic of Korea; 3 Division of Analytical Science, Korea Basic Science Institute, 169-148 Gwahak-ro, Yuseong-gu, Daejeon 34133 Republic of Korea; 4 Present Address: Research and Development Division, World Institute of Kimchi, 86 kimchi-ro, Nam-gu, Gwangju 61755 Republic of Korea

**Keywords:** Mechanisms of disease, Metabolic disorders

## Abstract

Browning of white adipose tissue **(**WAT) is currently considered a potential therapeutic strategy to treat diet-induced obesity. While some probiotics have protective effects against diet-induced obesity, the role of probiotics in adipose browning has not been explored. Here, we show that administration of the probiotic bacterium *Lactobacillus amylovorus* KU4 (LKU4) to mice fed a high-fat diet (HFD) enhanced mitochondrial levels and function, as well as the thermogenic gene program (increased *Ucp1*, *PPARγ*, and *PGC-1α* expression and decreased *RIP140* expression), in subcutaneous inguinal WAT and also increased body temperature. Furthermore, LKU4 administration increased the interaction between PPARγ and PGC-1α through release of RIP140 to stimulate *Ucp1* expression, thereby promoting browning of white adipocytes. In addition, lactate, the levels of which are elevated in plasma of HFD-fed mice following LKU4 administration, elicited the same effect on the interaction between PPARγ and PGC-1α in 3T3-L1 adipocytes, leading to a brown-like adipocyte phenotype that included enhanced *Ucp1* expression, mitochondrial levels and function, and oxygen consumption rate. Together, these data reveal that LKU4 facilitates browning of white adipocytes through the PPARγ-PGC-1α transcriptional complex, at least in part by increasing lactate levels, leading to inhibition of diet-induced obesity.

## Introduction

Obesity poses a major threat to human health since it is closely associated with the incidence of metabolic disorders, including diabetes and cardiovascular diseases^1^. Chronic over-nutrition coupled with low physical activity promotes fat accumulation in white adipose tissue (WAT), the main site of energy storage, and excessive expansion of WAT mass leads to obesity^2^. In contrast, brown adipose tissue (BAT) is specialized for energy dissipation. Brown adipocytes release energy in the form of heat by uncoupling oxidative phosphorylation from ATP production through uncoupling protein 1 (UCP1)-mediated leakage of protons from the intermembrane compartment into the mitochondrial matrix^3,4^. Therefore, enhancement of BAT function has promising potential as a therapeutic strategy to treat obesity and associated metabolic disorders. Recently, numerous studies have shown that thermogenic stimuli such as cold exposure or β-adrenergic stimulation can induce brown-like adipocytes (also known as beige or brite adipocytes) in subcutaneous inguinal WAT (iWAT). Beige adipocytes acquire the energy-releasing characteristics of BAT, efficiently protecting against diet-induced obesity by promoting energy expenditure^4,5^. Notably, following this environmental or hormonal stimulation, transcriptional regulators of BAT such as PGC-1α and PRDM16 are induced in WAT and coordinate with the key adipocyte transcription factor, PPARγ, to promote the expression of brown-selective genes; this leads to browning of white adipocytes in combination with repression of the expression or activities of negative regulatory factors like RIP140 and RB1 during the β-adrenergic-induced WAT browning^6,7^.

Probiotics are thought to exert various beneficial effects on human health^8^. We and other groups have recently shown that administration of probiotic bacteria to mice fed a high-fat diet (HFD) promotes fatty acid oxidation in metabolic tissues, reducing adiposity and thereby protecting against diet-induced obesity and its related metabolic disorders^9–12^. This suggests that this beneficial effect of probiotic bacteria may be associated with beige conversion of WAT; consequently, we hypothesized that some probiotics may promote or induce browning of white adipocytes, which, in turn, may facilitate energy expenditure and protect against diet-induced obesity. Here, we demonstrate that administration of *Lactobacillus amylovorus* KU4 (LKU4), a probiotic bacterium, to mice fed a HFD increased mitochondrial levels and expression of BAT-selective genes in iWAT, with a concomitant increase in body temperature; additionally, we also show that lactate mediates these effects of LKU4 on the browning of white adipocytes by remodeling the PPARγ transcription complex through switching RIP140 to PGC-1α, consequently leading to protection against HFD-induced obesity.

## Results

### LKU4 administration improved HFD-induced obesity

**Figure 1:**
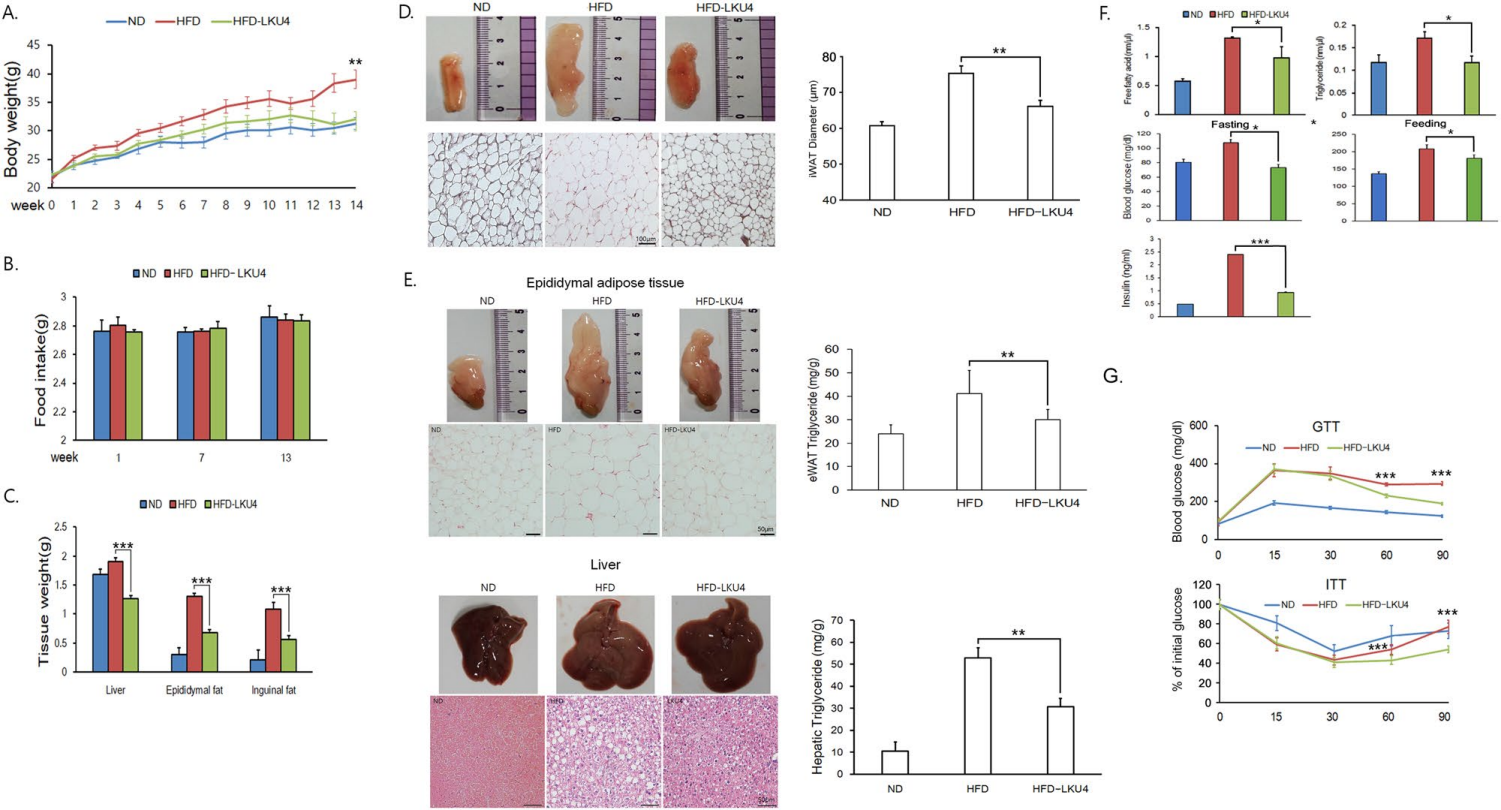
The effect of LKU4 administration on HFD-induced obesity and insulin resistance. LKU4 or PBS was administered daily to C57BL/6 male mice for 14 weeks during feeding with a ND or a HFD. Body weight (**A**), food intake (**B**), and tissue weight (**C**) of mice were measured as indicated (n = 8–10 per group). (**D**) Shown are representative images (left upper), histology (left middle), and adipocyte size (in diameter; lower) in iWAT from each group of mice (n = 160 adipocytes; 40 adipocytes x 4 animals per group). (**E**) Representative images (left upper), histology (left middle; n = 4 per group) and triglyceride content of the eWAT and the liver (lower; n = 6–9 per group). (**F**) Plasma levels of free fatty acids, triglycerides, glucose, and insulin (n = 5–8 per group). (**G**) Glucose tolerance and insulin tolerance tests were performed on each group of mice after 14 weeks of feeding (n = 6–8 per group). HFD *vs* HFD-LKU4; *P < 0.05, **P < 0.005, ***P < 0.001.

To determine whether LKU4 can ameliorate HFD-induced metabolic disorders such as obesity and insulin resistance, 7-week-old male mice were fed a HFD with or without oral administration of LKU4 for 14 weeks. Mice fed a HFD with LKU4 oral supplementation (HFD-LKU4 mice) showed a reduction in body weight gain (19%), as well as in liver (34%), epididymal (47%), and inguinal WAT weight (48%), compared to mice fed a HFD (HFD mice) (Fig. 1A,C), even though food intake was comparable between the two groups (Fig. 1B). Notably, the subcutaneous iWAT of HFD-LKU4 mice presented a reddish appearance and the adipocyte diameter in iWAT was reduced by 12% compared to the HFD mice (Fig. 1D). Consistently, adipocyte size in eWAT and lipid droplet deposition in liver also reduced in response to LKU administration in parallel with reduction of triglyceride levels in in these tissues as compared with HFD mice (Fig. 1E). Furthermore, the plasma levels of free fatty acids and triglycerides in HFD-LKU4 mice were reduced by 16% and 31%, respectively, compared to mice fed a HFD. In addition, fed and fasting plasma glucose levels and fasting insulin levels in HFD-LKU4 mice were reduced by approximately 11, 13, and 67%, respectively, relative to those in control HFD mice (Fig. 1F). Diet-induced obesity is closely associated with insulin resistance. Since LKU4 administration reduced diet-induced obesity and inhibited the increase in plasma glucose and insulin levels resulting from a HFD, we next conducted a glucose tolerance test (GTT) and an insulin tolerance test (ITT) to determine whether LKU4 could improve insulin sensitivity in mice fed a HFD. After intraperitoneal glucose injection, HFD and HFD-LKU4 mice showed similar plasma glucose levels after 15 min. However, while the plasma glucose levels of HFD-LKU4 mice decreased continuously and markedly until 90 min after glucose injection, the plasma glucose levels of HFD mice were not significantly reduced, even at 90 min after glucose injection (Fig. 1G). In the ITT, although the insulin-induced reduction in plasma glucose levels was not markedly different until 30 min after insulin injection, glucose levels in the HFD mice increased markedly 60 min after insulin injection. However, HFD-LKU4 mice continuously maintained reduced plasma glucose levels. Together, these results indicate that LKU4 administration confers resistance to HFD-induced obesity.

### LKU4 administration promoted a BAT-like phenotype in subcutaneous iWAT of HFD mice

**Figure 2:**
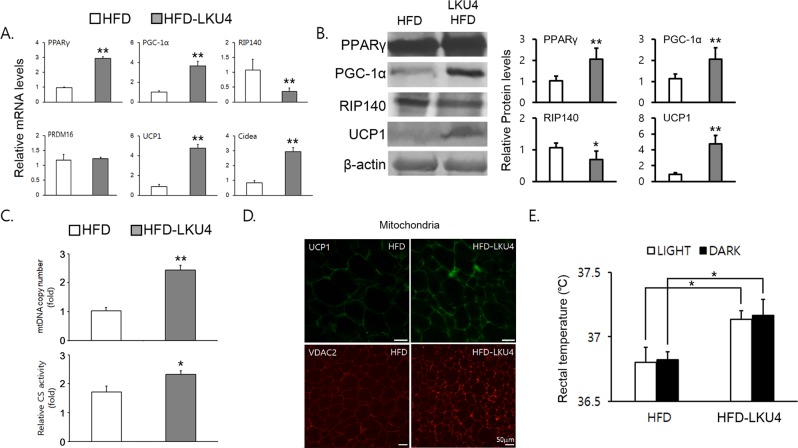
LKU4 administration induces browning of iWAT in mice fed a HFD. (**A**,**B**) The expression of browning genes in iWAT was determined by RT-qPCR (**A**) and immunoblotting (Cropped blots were used). (**B**–**D**) Mitochondrial DNA (mtDNA) copy number and citrate synthase (CS) activity (**C**) and representative images of VDAC2 and UCP1 staining (**D**) in iWAT of HFD and HFD-LKU4 mice. (**E**) Rectal temperatures were measured in each group of mice at week 12 (n = 8–10). HFD *vs* HFD-LKU4; *P < 0.05, **P < 0.005.

Recent studies have revealed that adipocytes in iWAT can be converted to brown-like adipocytes in response to cold exposure, β-adrenergic stimulation, and PPARγ agonist treatment^13^, and this process is considered to effectively protect against diet-induced obesity. To determine whether the anti-obesity effect of LKU4 on HFD mice is associated with browning of white adipocytes, we first analyzed BAT gene expression in subcutaneous iWAT. As shown in Fig. 2A, LKU4 administration distinctly elevated the mRNA levels of genes required for thermogenesis, such as *Ucp1* and *Cidea*, in iWAT of HFD mice. Concomitantly, the mRNA levels of *Pparγ* and *Pgc*-*1α*, key transcriptional regulators of adipocyte browning, were also increased. However, *Prdm16* mRNA levels were not significantly affected by LKU4 administration. In contrast, the mRNA levels of *Rip140*, which is known to inhibit adipocyte browning, were greatly reduced in iWAT of HFD mice due to LKU4 administration. Administration of LKU4 consistently increased UCP1, PPARγ, and PGC-1α proteins levels, while those of RIP140 were decreased (Fig. 2B). Administration of LKU4 also increased mitochondrial DNA (mtDNA) copy number and the activity of citrate synthase, an indicator of mitochondrial function (Fig. 2C). Furthermore, immunostaining of UCP1 and voltage-dependent anion channel 2 (VDAC2), a protein that is localized in the outer mitochondrial membrane, increased strongly in iWAT sections from HFD-LKU4 mice compared to HFD mice (Fig. 2D). Accordingly, higher core body temperatures were maintained in HFD-LKU4 mice than in HFD mice, both during the day and at night (Fig. 2E), suggesting that LKU4 administration enhances thermogenesis in HFD mice. These results strongly indicate that LKU4 administration is sufficient to induce the conversion of white to beige adipocytes in iWAT.

### LKU4-CM facilitated browning of 3T3-L1 adipocytes

**Figure 3:**
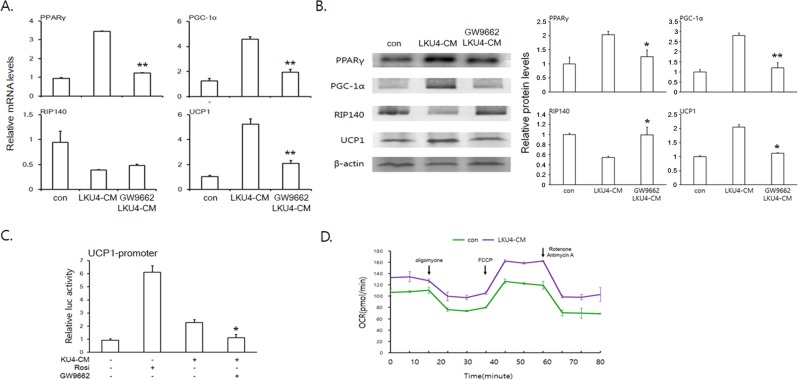
LKU4-CM induces browning of 3T3-L1 adipocytes. (**A**,**B**) Day 6 3T3-L1 adipocytes were incubated with or without LKU4-CM and GW9662 for 36 h, as indicated. The mRNA (**A**) and protein levels (**B**) of genes involved in adipocyte browning were analyzed by RT-qPCR and immunoblotting (Cropped blots were used), respectively, and the results were expressed as fold changes compared to the control. (**C**) Luciferase activity in HEK293T cells cotransfected with a reporter plasmid containing the *Ucp1* promoter (pGL3-*Ucp1*-Luc) and the pcDNA3-PPARγ and pCMX-RXRα expression plasmids. After 12 h of transfection, cells were incubated with LKU4-CM, rosiglitazone (Rosi, 5.5 μM), and/or GW9662 for 24 h, as indicated. (**D**) Day 8 3T3-L1 adipocytes were incubated with control bacterial culture medium (con) or LKU4-CM for 36 h and the OCR was then measured in the basal condition and in the presence of oligomycin, FCCP, and rotenone/antimycin A, at the indicated time point. LKU4-CM *vs* LKU4-CM + GW9662; *P < 0.05, **P < 0.005.

To further determine whether LKU4 is sufficient to induce adipocyte browning, 3T3-L1 preadipocytes were differentiated to adipocytes by adipogenic stimuli, following which day 6 3T3-L1 adipocytes were treated with LKU4-conditioned medium (LKU4-CM) for 36 h. Consistent with the *in vivo* results, LKU4-CM treatment increased both the mRNA and protein levels of UCP1, PPARγ, and PGC-1α while those of RIP140 were decreased (Fig. 3A,B). However, when GW9662, a PPARγ antagonist, was added to 3T3-L1 adipocytes in combination with LKU4-CM, the effect of LKU4 on the expression of these genes was dramatically reduced. Since GW9662 inhibited the LKU4-CM-induced expression of *Ucp1*, a key PPARγ target gene in beige adipocytes, we performed a luciferase reporter assay to test whether the browning effect of LKU4-CM on white adipocytes was achieved through stimulation of PPARγ activity. Interestingly, treatment with both rosiglitazone, a PPARγ agonist, and LKU4-CM increased the relative activity of luciferase when driven by the mouse *Ucp1* promoter (−2620 to +68 bp) approximately 6.2- and 2.3-fold, respectively, in HEK293T cells. However, co-treatment with GW9662 abolished the enhancing effect of LKU4-CM on the activity of the *Ucp1* promoter (Fig. 3C). Next, we evaluated the effect of LKU4-CM on the metabolic rate of adipocytes. As shown in Fig. 3D, the basal and maximal oxygen consumption rates (OCRs) of LKU4-CM-treated 3T3-L1 adipocytes were both significantly higher than those of the control 3T3-L1 adipocytes. These results indicate that LKU4-CM induces browning of 3T3-L1 adipocytes and increases energy expenditure.

### LKU4 enhanced PPARγ activity by changing the PGC-1α to RIP140 ratio associated with PPARγ

**Figure 4:**
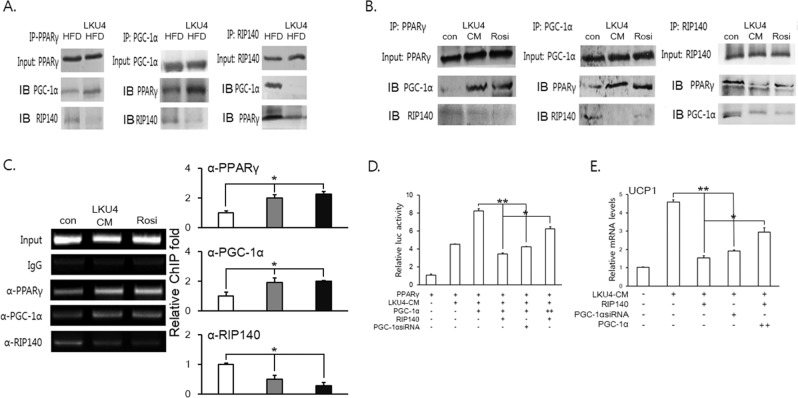
LKU4 facilitates the interaction between PPARγ and PGC-1α in iWAT and 3T3-L1 adipocytes, which stimulates *Ucp1* expression. (**A**) IP analysis of the interaction between PPARγ, PGC-1α, and RIP140 in iWATs of HFD- and HFD-LKU4-fed mice using the indicated antibodies (Cropped blots were used). (**B**) Day 8 3T3-L1 adipocytes cotransfected with PPARγ, PGC-1α, and RIP140 expression plasmids were treated with LKU4-CM or rosiglitazone (5.5 μM) for 24 h, following which IP analysis was performed with the indicated antibodies (Cropped blots were used). (**C**) A ChIP assay was performed on day 7 3T3-L1 adipocytes cotransfected with PPARγ, PGC-1α, and RIP140 expression plasmids in the presence or absence of LKU4-CM and rosiglitazone (5.5 μM). Protein/DNA complexes were immunoprecipitated with the indicated antibodies and the *Ucp1* promoter region containing a PPRE (−2551 to −2541 bp) was PCR-amplified from immunoprecipitated DNA. Con *vs* LKU4-CM; *P < 0.05 (**D**) Luciferase activity in HEK293T cells cotransfected with a reporter plasmid (pGL3-*Ucp1-*Luc) and the indicated expression plasmids and/or siRNA. After 12 h of transfection, cells were treated either with control medium or LKU4-CM for 48 h. (**E**) Day 6 3T3-L1 adipocytes were transfected with the indicated expression plasmids and/or siRNA. After 12 h of transfection, cells were incubated in the presence or absence of LKU4-CM for 48 h and harvested for RT-qPCR analysis. *P < 0.05, **P < 0.005.

Our results showed that both LKU4 and LKU4-CM increase PGC-1α and decrease RIP140 levels, *in vivo* and *in vitro*. Furthermore, RIP140 functions as a co-repressor of *PPARγ* and has been documented to interact with both the PPARγ and PGC-1α proteins. To determine whether LKU4 enhances browning of white adipocytes by regulating PPARγ activity, i.e., by eliciting changes in the ratio of RIP140 to PGC-1α in the PPARγ transcriptional complex, we first performed immunoprecipitation (IP) experiments in iWAT of HFD mice. When iWAT lysates were immunoprecipitated with an anti-PPARγ antibody, LKU4 administration strongly induced the recruitment of PGC-1α to PPARγ, with subsequent inhibition of RIP140-PPARγ interaction. Accordingly, the results of the IP assay using an anti-PGC-1α antibody showed increased interaction between PGC-1α and PPARγ in iWAT of HFD-LKU4 mice, whereas PGC-1α-RIP140 interaction was inhibited. Furthermore, IP with an anti-RIP140 antibody showed that LKU4 administration reduced the recruitment of PGC-1α and PPARγ to RIP140 (Fig. 4A). We then performed *in vitro* IP on 3T3-L1 adipocytes treated with LKU4-CM or rosiglitazone using anti-PPARγ, ant-PGC-1α, and anti-RIP140 antibodies. Consistent with the *in vivo* IP results, LKU4-CM and rosiglitazone treatment both increased the interaction between PGC-1α and PPARγ in parallel with inhibition of RIP140 interaction with either PPARγ or PGC-1α (Fig. 4B). The results indicated that LKU4 removes RIP140 from the PPARγ transcriptional complex, concomitant with PGC-1α recruitment. Next, we performed ChIP assays on day 8 3T3-L1 adipocytes to determine whether LKU4-CM affects the recruitment of PPARγ, PGC-1α, and RIP140 to the mouse *Ucp1* promoter region containing a PPAR response element (PPRE). As shown in Fig. 4C, treatment with either rosiglitazone or LKU4-CM increased PPARγ binding to the *Ucp1* promoter containing the PPRE, with increased PGC-1α occupancy, while RIP140 recruitment to this promoter region was strongly reduced by both treatments. These data suggest that LKU4 may evoke a switch from a repressive RIP140-PPARγ complex to a stimulatory PGC-1α-PPARγ complex in the *Ucp1* promoter region, thereby facilitating *Ucp1* expression. To further confirm that LKU4 regulates PPARγ activity by modulating the relative PGC-1α to RIP140 ratio, we performed a luciferase assay in HEK293T cells using a luciferase reporter fused to the *Ucp1* −2620 to +68 bp promoter region (pGL3*-Ucp1*-Luc). As shown in Fig. 4D, addition of LKU4-CM significantly increased PPARγ transactivation of the *Ucp1* promoter in HEK293T cells. When PGC-1α was added together with PPARγ, LKU4-CM further increased *Ucp1* promoter activity. However, this promoting effect of PGC-1α on PPRAγ-induced *Ucp1* promoter activity was inhibited by RIP140 and this repressive effect of RIP140 was partially inhibited by the addition of PGC-1α. The RT-qPCR analysis showed that treatment of day 8 3T3-L1 adipocytes with LKU4-CM markedly increased *Ucp1* mRNA levels. However, when either PGC-1α siRNA or RIP140 was transfected into 3T3-L1 adipocytes, the inducing effect of LKU4-CM on *Ucp1* expression was reduced by approximately 57% and 65%, respectively. In contrast, when 3T3-L1 adipocytes were cotransfected with RIP140 and high concentrations of PGC-1α, the RIP140-mediated inhibition of LKU4-CM-induced *Ucp1* expression was partially released, suggesting that LKU4 may increase *Ucp1* expression in adipocytes by enhancing PPARγ activity through increased interaction between PGC-1α and PPARγ (Fig. 4E).

### Lactate is a key LKU4 metabolite that increases the expression of genes important for adipocyte browning

**Figure 5:**
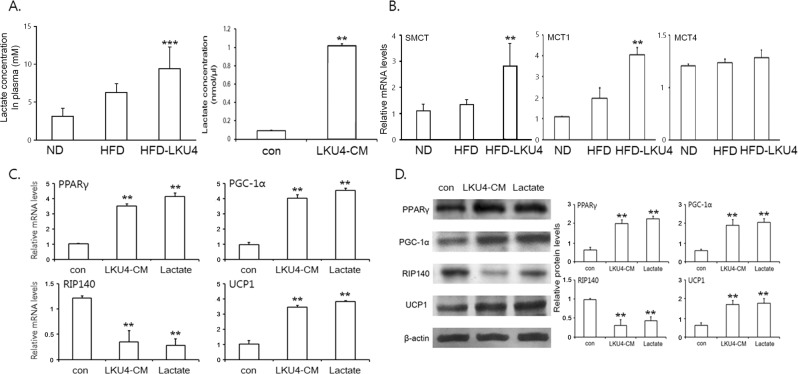
LKU4 increases lactate levels in HFD-fed mice and 3T3-L1 adipocytes. (**A**) Lactate concentrations were measured in plasma from each group of mice (left) and in the media (bacterial culture medium; con and LKU4-CM) (right). HFD *vs* HFD-LKU4; ***P < 0.001, con *vs* LKU-CM; **P < 0.005 (**B**) RT-qPCR analysis of *Smct* mRNA levels in the intestine and *Mct1* and *Mct4* mRNA levels in the iWAT from each group of mice. HFD *vs* HFD-LKU4; **P < 0.005 (*C* and *D*) Day 8 3T3-L1 adipocytes were incubated with control bacterial culture medium (con), LKU4-CM, or 5 mM lactate for 36 h and the mRNA (**C**) and protein levels (**D**) of genes involved in adipocyte browning were analyzed by RT-qPCR and immunoblotting (Cropped blots were used), respectively. con *vs* LKU4-CM or Lactate; **P < 0.005.

Accumulating evidences suggest that adipose browning is affected by the gut microbial communities and many *Lactobacillus* species have shown to alter the gut microbiota^2,14^. Interestingly, a recent study showed that every-other-day fasting alters the gut microbial communities (increase of *Firmicutes* with decrease of *Bacteroidetes* and *Proteobacteria*), which increases the levels of plasma lactate, leading to adipocyte browning in iWAT^15^. Consistently, LKU4 administration increased *Lactobacillus* species (the phylum *Firmicutes*) with decrease of *Enterobacteriaceae* (the phylum *Protobacteria*) in feces (Supplementary Information Fig. 1). In addition, we were also able to confirm viable LKU4 cells in feces (5.5 × 10^3^ cfu/mg feces) (data not shown). Next, we analyzed whether LKU4 administration could elevate plasma lactate levels in HFD mice. As shown in Fig. 5A, when LKU4 was administered during HFD feeding, plasma lactate levels were approximately 1.5-fold higher than without LKU4 administration. In addition, lactate levels in LKU4-CM were 5-fold higher than those in control media. Furthermore, LKU4 administration increased not only the mRNA levels of sodium-lactate-mediated transporter (SMCT) in the intestine and but also the mRNA levels of proton-linked monocarboxylate transporter 1 (*Mct1*) in iWAT of HFD mice (Fig. 5B). However, LKU4 administration did not affect *Mct4* expression in iWAT of HFD mice. Lactate is a key metabolite of the gut microbiota that is utilized in the gut and also can be released into systemic circulation via SMCT^16^. Moreover, MCT1 is responsible for lactate transport across the plasma membrane of adipocytes and its expression is regulated by stimuli for adipocyte browning^12,17^. Therefore, our data suggest that increased production of lactate by LKU4 feeding is released into the circulation and taken up to adipocytes. To determine whether the promoter activity and expression of *Ucp1* gene are affected by lactate in a dose-dependent manner, we performed a repoter gene assay in HEK293T cells using pGL3*-Ucp1*-Luc and RT-qPCR assay in 3T3-L1 adipocytes, resepectively. As shown in Supplementary Information Fig. 2, the promoter activity and expression of UCP1 were not dramatically changed in 2.5 mM lactate. However, 5 mM lactate increased the promoter activity and expression of UCP1 gene by approximately 3.9 and 3.7-fold, respectively, relative to untreated cells and 10 mM lactate showed the similar effect with 5 mM on the promoter activity and expression of UCP1.

To further test whether lactate could mediate LKU4-induced iWAT browning, we first compared the effect of LKU4-CM and lactate treatments on the expression of genes involved in adipocyte browning. Consistent with the results of LKU4-CM treatment, the mRNA levels of *Ucp1*, *PPARγ*, and *PGC-1α* were increased in 3T3-L1 adipocytes following 5 mM lactate treatment (Fig. 5C). In contrast, lactate treatment decreased the mRNA levels of the *RIP140* gene. As expected, lactate treatment also increased UCP1, PPARγ, and PGC-1α protein levels in 3T3-L1 adipocytes by 1.7-, 2.2-, and 2-fold, respectively, relative to control adipocytes, whereas RIP140 protein levels were reduced by 60% (Fig. 5D). Furthermore, knockdown of MCT1 by MCT1-specific siRNAs reduced the expression of *Ucp1* gene by 47~51% of those in LKU4-CM-treated adipocytes, suggesting that lactate is essential for LKU4-CM-induced browning of adipocytes (Supplementary Information Fig. 3).

### Lactate facilitated browning of 3T3-L1 adipocytes by modulating the interaction of PGC-1α and RIP140 with PPARγ

**Figure 6:**
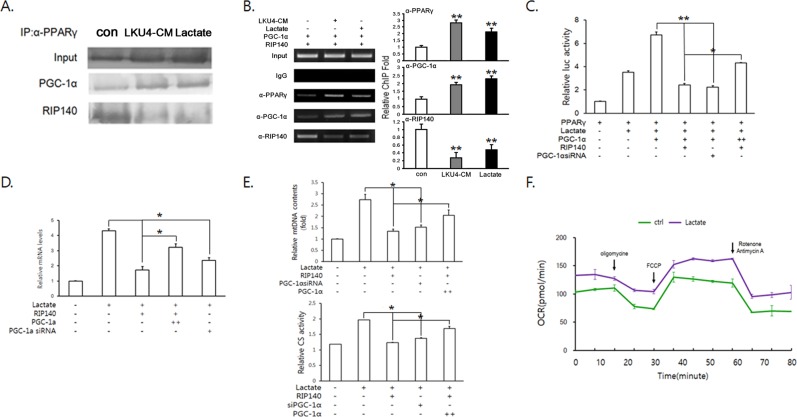
Lactate induces browning of 3T3-L1 adipocytes by enhancing PPARγ activity. (**A**) IP analysis using an anti-PPARγ antibody in day 9 3T3-L1 adipocytes cotransfected with *PPARγ, PGC-1α*, and *RIP140* expression plasmids following 24 h treatment with control bacterial culture medium (con), LKU4-CM, or 5 mM lactate (Cropped blots were used). (**B**) ChIP assays using the indicated antibodies or normal IgG in day 7 3T3-L1 adipocytes cotransfected with *PGC-1α* and *RIP140* expression plasmids in the presence or absence of LKU4-CM or 5 mM lactate, as indicated. The *Ucp1* promoter containing a PPRE was PCR-amplified from immunoprecipitates. con *vs* LKU4-CM, Lactate; **P < 0.005 (**C**) Relative *Ucp1* promoter activity in HEK293T cells cotransfected with pGL3-*Ucp1*-Luc and indicated expression plasmids or siRNA in the presence or absence of lactate (5 mM). (**D**,**E**) RT-qPCR analysis of *Ucp1* (**D**) and mitochondrial DNA (mtDNA) copy number and citrate synthase (CS) activity (**E**) in day 8 3T3-L1 adipocytes cotransfected with the indicated expression plasmids or siRNA after 48 h of treatment with vehicle or lactate. (**F**) Day 8 3T3-L1 adipocytes were incubated with vehicle (ctrl) or lactate for 24 h and the OCR was then measured in the basal condition and in the presence of oligomycin, FCCP, and rotenone/antimycin A at the indicated time point (**C**–**E** *P < 0.05, **P < 0.005).

Next, we performed an IP assay using an anti-PPARγ antibody to determine whether lactate could also affect the interaction of PGC-1α and RIP140 with PPARγ. Consistent with that observed with LKU4-CM treatment, lactate also facilitated complex formation between PPARγ and PGC-1α, in parallel with reduced interaction between PPARγ and RIP140 (Fig. 6A). Furthermore, ChIP assays also showed that lactate and LKU4-CM treatments both increased PPARγ binding to the *Ucp1* promoter region containing the PPRE, with increased PGC-1α occupancy. In contrast, lactate treatment reduced RIP140 recruitment to this *Ucp1* promoter region (Fig. 6B). Furthermore, lactate promoted the PPARγ-mediated induction of luciferase reporter (pGL3*-Ucp1-Luc*) activity in HEK293T cells and PGC-1α further enhanced lactate-mediated PPARγ transcriptional activity (Fig. 6C). When RIP140 or siRNA targeting PGC-1α was cotransfected with PGC-1α into HEK293T cells, the promotion of PPARγ activity by lactate and PGC-1α was significantly suppressed. However, addition of increased PGC-1α levels partially inhibited this suppressive effect of RIP140 on PPARγ activity. Consistent with these results, RT-qPCR analysis also showed that lactate treatment led to increased *Ucp1* expression and this inducing effect of lactate on *Ucp1* expression was suppressed by either RIP140 or siRNA targeting PGC-1α (Fig. 6D). When PGC-1α was added to 3T3-L1 adipocytes transfected with RIP140, the suppressive effect of RIP140 on lactate-induced *UCP1* expression was partially inhibited. Next, we analyzed the effect of lactate on mitochondrial levels and activity in 3T3-L1 adipocytes in relation to these co-regulators. As shown in Fig. 6E, mtDNA copy number and citrate synthase activity were increased following lactate treatment, indicating that lactate enhances mitochondrial biogenesis. When RIP140 or siRNA targeting PGC-1α was transfected, the effects of lactate on mtDNA copy number and citrate synthase activity were strongly inhibited. However, when PGC-1α was cotransfected with RIP140 into 3T3-L1 adipocytes, these effects of lactate on mitochondrial biogenesis and function were partially rescued. In addition, lactate treatment increased basal and maximal OCR levels in 3T3-L1 adipocytes, consistent with the effect of LKU4-CM on the OCR (Fig. 6F). These results strongly indicate that lactate mediates the effect of LKU4 on the conversion of white adipocytes to BAT-like adipocytes, at least in part.

## Discussion

Metabolic imbalance due to high energy intake and low energy expenditure causes obesity and its associated metabolic complications^18^. Recently, induction of a brown adipocyte-like phenotype in iWAT has been considered as a potential therapeutic approach for the treatment of diet-induced obesity by promoting energy expenditure^5^. Here, we demonstrated that administration of LKU4, a probiotic bacteria, to HFD mice resulted in increased mitochondrial levels and activity and enhanced the expression of BAT-specific genes such as *Ucp1* and *Cidea* in iWAT; this, in turn, activated adipocyte browning and enhanced body temperature, resulting in protection from diet-induced obesity. Consistent with the *in vivo* results, *in vitro* LKU4-CM treatment elicited the same effect on mitochondrial biogenesis and BAT gene expression, leading to increased OCRs in 3T3-L1 adipocytes.

The RIP140 protein has been reported to interact with a variety of transcription factors, especially nuclear receptors, that play key roles in metabolic homeostasis^19^. Moreover, RIP140 is highly expressed in metabolic tissues, including WAT. Mice null for RIP140 show resistance to fat accumulation in WAT and diet-induced obesity^20^. In the absence of RIP140, the expression of BAT-specific genes like *Ucp1* in WAT is enhanced, along with oxygen consumption^7^. In this study, we observed that RIP140 expression in iWAT of HFD mice was significantly downregulated following LKU4 administration, which was accompanied by increased expression of *Ucp1* and *Cidea*. These genes are also known targets of the coactivator PGC-1α. The PGC-1α protein is known to induce mitochondrial biogenesis and energy dissipation as heat, processes that are important for the brown adipocyte phenotype^17^. Recent studies have shown that PGC-1α is induced in subcutaneous WAT in response to thermogenic stimuli such as β-adrenergic signaling and cold exposure and promotes the conversion of white adipocytes to beige adipocytes by stimulating thermogenic gene expression in cooperation with a key transcription factor, PPARγ^21^. Interestingly, LKU4 administration increased PGC-1α expression, both in iWAT of HFD mice and in 3T3-L1 adipocytes, and facilitated physical interaction between PGC-1α and PPARγ, both *in vivo* and *in vitro*, stimulating PPARγ transcriptional activity. We found that interaction between PGC-1α and PPARγ increased in parallel with a reduced affinity of RIP140 for PPARγ. Consistent with this result, the ChIP assay also showed that LKU4 enhanced PPARγ and PGC-1α recruitment to the *Ucp1* promoter region containing the PPRE, whereas RIP140 recruitment to this promoter region was reduced. Accordingly, LKU4-mediated induction of PPARγ transcriptional activity and *Ucp1* expression was reduced by RIP140 or by silencing of PGC-1α in 3T3-L1 adipocytes and overexpression of PGC-1α partially rescued the RIP140-induced inhibition of these LKU4 effects. Our data suggest that the browning effect of LKU4 on iWAT can be attributed to remodeling of the PPARγ transcriptional complex in a manner favorable for the expression of BAT-specific genes such as *Ucp1*.

Recent studies revealed that gut microorganisms have important roles in host physiology including host immunity and energy metabolism^2,14,22^. A variety of intermediate metabolites produced by these microorganisms functions as signaling molecules in these events^22,23^. Therefore, the composition of the gut microbiota and their metabolites is closely associated with host physiology including energy homeostasis. Although we did not determine all the phylum of the gut microbiota, LKU4 administration increased *Lactobacillus* species (the phylum *Firmicutes*) with decrease of *Enterobacteriaceae* (the phylum *Protobacteria*) in the gut; moreover, this change was similar to the effects of every-other-day fasting on the gut microbiota. The alterations in the gut microbiota due to every-other-day fasting have been shown to play a key role in adipose browning via increased plasma lactate levels and increased expression of MCT1 in adipocytes^15^. In addition, exercise also increases plasma lactate to induce BAT-like adipocytes in WAT; however, the manner in which lactate facilitates adipocyte browning remains mostly unexplored^24^. Lactate produced by the gut bacteria, primarily by *Lactobacillus*, is utilized in the gut and also released into the circulation through SMCT^25^. Lactate is transported across the membrane of white adipocytes by MCT1, which is induced in mouse iWAT following cold exposure^26^. In the present study, intestinal SMCT, plasma lactate and iWAT MCT1 levels in HFD mice were increased by LKU4 administration. Consistent with administration of LKU4 to HFD mice and LKU4-CM treatment to 3T3-L1 adipocytes, lactate enhanced the interaction between PPARγ and PGC-1α and reduced the affinity of RIP140 for PPARγ; in turn, this facilitated PPARγ and PGC-1α recruitment to the *Ucp1* promoter region in 3T3-L1 adipocytes. Furthermore, lactate induction of 3T3-L1 adipocyte browning was inhibited either by RIP140 or by silencing of PGC-1α, suggesting that modulation of the PPARγ transcriptional complex by LKU4-induced lactate facilitates browning of white adipocytes in WAT, which, in turn, inhibits diet-induced obesity in HFD mice. Although LKU4-induced increase of plasma lactate levels is not as high as exercise, long-term administration of LKU4 may continuously stimulate to open the browning program and eventually lead to browning of adipocytes. Taken together, our findings show that LKU4 administration facilitates browning of iWAT by promoting PPARγ-PGC-1α interaction by increasing lactate levels, at least in part, which ameliorates diet-induced obesity in mice. This indicates that LKU4 may provide a therapeutic platform to inhibit diet-induced obesity and associated metabolic disturbances by increasing energy expenditure.

## Materials and Methods

All experiments were performed in accordance with relevant guidelines and regulations.

### Animal experimentation

All animal studies were performed according to procedures approved by the Institutional Animal Care and Use Committee of Chonnam National University. C57BL/6 male mice (7 weeks old; weight, 19 ± 2 g; Damul Science, Daejeon, Korea) were fed either a normal diet (ND; 16% of the total calories obtained from fat, Damul Science) or a HFD (45% of the total calories obtained from fat, Research Diets Inc., New Brunswick, USA) under a 12-h light/dark cycle. LKU4 cells were cultured in MRS broth and resuspended in PBS at ~1.0 × 10^8^ cfu/mL and 200 μL of this LKU4 resuspension, or PBS, was orally administrated daily to the mice for 14 weeks.

### Cell culture, transfection and preparation of LKU4-CM

HEK293T and 3T3-L1 cells were cultured in DMEM containing 10% fetal bovine serum or newborn calf serum and antibiotics. Plasmids pcDNA3-*PPARγ*, pCMX-*RXRα*, pcDNA3-*PGC-1α*, and pEF-*RIP140* were previously described^27,28^. The mouse *Ucp1-luciferase* reporter plasmid (3.1 kb) was provided by Dr. Robert A. Kozak^29^. The *Ucp1* promoter (−2620 to +68 bp) was PCR-amplified from 3T3-L1 genomic DNA and cloned into the pGL3 vector (Promega, Madison, WI, USA) to generate pGL3*-Ucp1*. Adipocyte differentiation of 3T3-L1 cells and transfections were performed as previously described^9,30^. Short interfering RNA (siRNA) targeting mouse PGC-1α was purchased from Bioneer (Daejeon, Korea). LKU4 cells grown to 0.6 at OD600 in MRS broth were pelleted down by centrifugation at 5,000 g for 15 min at 4 °C. The cell-free supernatant was used as a LKU4-CM at 1/100 volume of the media and LKU4-CM did not affect the pH of culture medium at this dilution. Homogenized fecal samples were serially diluted in anaerobic diluent (33 mM KH_2_PO_4_, 1.4 mM Na_2_HPO_4_, 0.05% L-cysteine, and 0.05% Tween 80; pH 7.2) and then, aliquots of the appropriate dilutions were spread onto the agar plates. Anaerobic bacteria were enumerated on GAM agar (Nissui Co., Tokyo, Japan) and plates were grown for 48 h in an anaerobic chamber (Concept 400, Ruskinn Technology, Leeds, UK). *Enterobcateriaceae* was enumerated according to the method by Itoh *et al*. using DHL agar and incubated at 37 °C for 24 h^31^. *Lactobacillus* species were enumerated on Lactobacillus-selection agar (LBS; BD, Heidelberg, Germany) and incubated at 37 °C for 48 h. *L. amylovorus* KU4 was selectively enumerated on Lactobacillus-selection agar (BD, Heidelberg, Germany) modified by the addition of nalidixic acid (800 µg/ml), neomycin sulfate (100 µg/ml), and vancomycin hydrosulfate (2.5 µg/ml) and incubated at 37 °C for 48 h.

### Immunohistochemistry

For histological analysis, tissues were fixed with a 10% formalin solution (Sigma-Aldrich, St. Louis, USA) in PBS (pH 7.4), and then embedded in paraffin. After serial dehydration, the sectioned tissues were stained with hematoxylin and eosin (H&E). To evaluate adipocyte size, the long and short axes were measured and averaged. Numerical data obtained using ImageJ are presented as means ± S.E.M. For immunohistochemistry on tissue samples, formalin-fixed tissues were frozen-sectioned. Anti-UCP1 (SH2436525, 1:500, Invitrogen, Carlsbad, USA) and anti-VDAC2 (ab37985, 1:200, AbCam, Cambridge, UK) antibodies were used as primary antibodies. Alexa 488-conjugated goat anti-rabbit (1:500) and Alexa 568-conjugated donkey anti-goat (1:1000) antibodies were used as secondary antibodies, respectively. Nuclei were counterstained with DAPI.

### RT-qPCR, ChIP, immunoblot, and immunoprecipitation assays

Total RNA extracted from cultured cells or tissues was reverse-transcribed to cDNA using M-MLV Reverse Transcriptase (Promega, Madison, WI, USA). The RT-qPCR analysis was performed as previously described^9^ and 36B4 RNA levels were used as a control for quantification of each gene. The ChIP assays were performed using normal IgG and anti-PPARγ, anti-PGC-1α, and anti-RIP140 antibodies in 3T3-L1 adipocytes. Primer sequences for PCR are listed in Supplementary Information Table 1. Immunoblotting was performed using anti-PPARγ, anti-PGC-1α, anti-RIP140, anti-UCP1, and anti-β-actin antibodies, as previously described^9^. For the IP assays, tissues and 3T3-L1 adipocytes were lysed in RIPA buffer by rotating incubation at 4 °C for 20 min. After a brief sonication, the lysates were immunoprecipitated using anti-PPARγ, anti-PGC-1α, and anti-RIP140 antibodies. Immunoprecipitates were subsequently analyzed by immunoblotting.

### Plasma and tissue analyses

The plasma levels of glucose, insulin, and lactate were measured using a glucose meter (OneTouch, LifeScan, Milpitas, USA), an insulin ELISA kit (ALPCO, Salem, NH, USA), and a lactate assay kit (Biovision, Milpitas, USA), respectively, according to the manufacturers’ recommended protocols. The triglyceride levels of tissue (liver, iWAT and eWAT) were measured using a triglyceride assay kit (Biovision, Milpitas, USA) according to the manufacturers’ recommended protocol. After 16 h of fasting, a glucose tolerance test (GTT) was performed in mice by intraperitoneal glucose injection (1 g/kg body weight). For the insulin tolerance test (ITT), insulin was injected intraperitoneally at 0.75 U/kg body weight. The blood glucose level was measured at 0, 15, 30, 60, and 90 min after glucose or insulin administration.

### Analyses of mitochondrial DNA, citrate synthase activity, and oxygen consumption rate

Mitochondrial DNA (mtDNA) was isolated from iWAT and 3T3-L1 adipocytes using a Mitochondrial Isolation kit (Biovision, Milpitas, USA) and qPCR was performed to determine mtDNA copy number using mtDNA primers (Supplementary Information Table 1). Citrate synthase activity was measured in iWAT and 3T3-L1 adipocytes using a citrate synthase activity assay kit (Biovision, Milpitas, USA) according to the manufacturer’s instructions. For measurement of OCR, differentiated 3T3-L1 adipocytes were seeded onto XFp cell culture microplates (Seahorse Bioscience, North Billerica, USA) containing 80 μL of growth medium (Seahorse Bioscience, North Billerica, USA) and incubated in a CO_2_ incubator at 37 °C for 24 h. A Seahorse Bioscience XFp analyzer was used to assess changes in dissolved oxygen levels in the media. All procedures followed the protocols recommended by the manufacturers.

### Statistical analysis

The Student’s *t*-test was used for statistical analyses of the data. The data were expressed as means ± S.D. or S.E. All the experiments were performed at least in triplicate. ANOVA test was used to distinguish the level of significance.

## Supplementary information

Supplementary Information 

## Data Availability

The datasets generated during and/or analyzed during the current study are available from the corresponding author on reasonable request.
