# Supplementary material for: Lactobacillus amylovorus KU4 ameliorates diet-induced obesity in mice by promoting adipose browning through PPARγ signaling
Source: Sci Rep. 2019 Dec 27;9:20152. doi: 10.1038/s41598-019-56817-w (PMC6934708; doi:10.1038/s41598-019-56817-w)
Supplement: Supplementary file 1 — Supplementary Information [file 41598_2019_56817_MOESM1_ESM.docx]

**Supplementary information**

***Lactobacillus amylovorus* KU4 ameliorates diet-induced obesity in mice by promoting adipose browning through PPARγ signaling**

Sung-Soo Park^1, 4^, Yeon-Joo Lee^1^, Hyuno Kang^3^, Garam Yang^1^, Eun Jeong Hong^1^, Jin Yeong Lim^3^, Sejong Oh^2*^, and Eungseok Kim^1*^

^1^Department of Biological Sciences, College of Natural Sciences, Chonnam National University, 77 Yongbong-ro, Buk-gu, Gwangju 61186, Republic of Korea

^2^Division of Animal Science, College of Agriculture & Life Science, Chonnam National University, 77 Yongbong-ro, Buk-gu, Gwangju 61186, Republic of Korea

^3^Gwangju Center, Korea Basic Science Institute D-15 Building, 77 Yongbong-Ro, Buk-Gu, Gwangju 61186, Republic of Korea

^4^Research and Development Division, World Institute of Kimchi, 86 kimchi-ro, Nam-gu, Gwangju 61755, Republic of Korea; present address

^1*^Correspondence: Department of Biological Sciences, College of Natural Sciences, Chonnam National University, 77 Yongbong-ro, Buk-gu, Gwangju 61186, South Korea.

E-mail: ekim@jnu.ac.kr, Tel: +82 62 530 3402, Fax: +82 62 530 3409

^2*^Correspondence: Division of Animal Science, College of Agriculture & Life Science, Chonnam National University, 77 Yongbong-ro, Buk-gu, Gwangju, 61186, South Korea.

E-mail: soh@jnu.ac.kr, Tel: +82 62 530 2116, Fax: +82 62 530 2129

Supplementary Figure 1. Number of viable fecal microorganisms in feces from each group of mice (n=6–8 per group). HFD *vs* HFD-LKU4; **P* < 0.05

Supplementary Figure 2. Dosage effect of lactate on the promoter activity and the expression of UCP1 gene. (A-B) Relative luciferase activity in HEK293T cells transfected with a reporter plasmid (pGL3-*Ucp1-*Luc) (A) and RT-qPCR analysis of *Ucp1* gene in 3T3-L1 adipocytes (B) after treatment of different concentration of lactate for 36 hr, as indicated. **P* < 0.05, ***P* < 0.01, ****P* < 0.001

Supplementary Figure 3. The silencing effect of MCT1 on LKU4-CM-induced *Ucp1* expression in 3T3-L1 adipocytes. (A) Silencing of *Mct1* gene by MCT1 specific siRNAs was assessed in 3T3-L1 adipocytes by RT-qPCR (B) MCT1 was silenced in 3T3-L1 adipocytes by transfection of two different MCT1 siRNAs and then the effect of LKU4-CM on the expression of *Ucp1* gene was assessed by RT-qPCR, as indicated. ****P* < 0.001

Supplementary Figure 4. GelDoc images of immunoblots showing expression of PPARγ, PGC-1α, RIP140, UCP1 and β-actin in iWAT of HFD and HFD-LKU4 mice in Figure 2B. After transferring the proteins from the gels to the PVDF membranes, membranes were cut and then immunoblotted. Unless otherwise indicated, each immunoblot is from different gels using the same tissue samples. Immunoblots from the same gel are in boxes. Representative bands shown in Figure 2B are marked with *.

Supplementary Figure 5. GelDoc images of immunoblots showing expression of PPARγ, PGC-1α, RIP140, UCP1 and β-actin in 3T3-L1 adipocytes in Figure 3B. After transferring the proteins from the gels to the PVDF membranes, membranes were cut and then immunoblotted. Unless otherwise indicated, each immunoblot is from different gels. Immunoblots from the same gel are in boxes. Representative bands shown in Figure 3B are marked with *.

Supplementary Figure 6. GelDoc images of immunoblots showing immunoprecipitation of PPARγ, PGC-1α, and RIP140 that shows the interaction between PPARγ, PGC-1α, and RIP140 in iWATs of HFD and HFD-LKU4 mice in Figure 4A. After transferring the proteins from the gels to the PVDF membranes, membranes were cut and then immunoblotted. Unless otherwise indicated, each immunoblot is from different gels using the same tissue samples. Immunoblots from the same gel are in boxes. Representative bands shown in Figure 4A are marked with *.

Supplementary Figure 7. GelDoc images of immunoblots showing immunoprecipitation of PPARγ, PGC-1α, and RIP140 that shows the interaction between PPARγ, PGC-1α, and RIP140 in 3T3-L1 adipocytes treated with LKU4-CM or rosiglitazone in Figure 4B. After transferring the proteins from the gels to the PVDF membranes, membranes were cut and then immunoblotted. Unless otherwise indicated, each immunoblot is from different gels. Immunoblots from the same gel are in boxes. Representative bands shown in Figure 4B are marked with *.

Supplementary Figure 8. GelDoc images of immunoblots showing expression of PPARγ, PGC-1α, RIP140, UCP1 and β-actin in 3T3-L1 adipocytes in Figure 5D. After transferring the proteins from the gels to the PVDF membranes, membranes were cut and then immunoblotted. Unless otherwise indicated, each immunoblot is from different gels. Immunoblots from the same gel are in boxes. Representative bands shown in Figure 5D are marked with *.

Supplementary Figure 9. GelDoc images of immunoblots showing PPARγ immunoprecipitation that shows the interaction between PPARγ, PGC-1α, and RIP140 in 3T3-L1 adipocytes treated with LKU4-CM or lactate in Figure 6A. After transferring the proteins from the gels to the PVDF membranes, membranes were cut and then immunoblotted. Unless otherwise indicated, each immunoblot is from different gels. Immunoblots from the same gel are in boxes. Representative bands shown in Figure 6A are marked with *.

Supplementary Figure 1


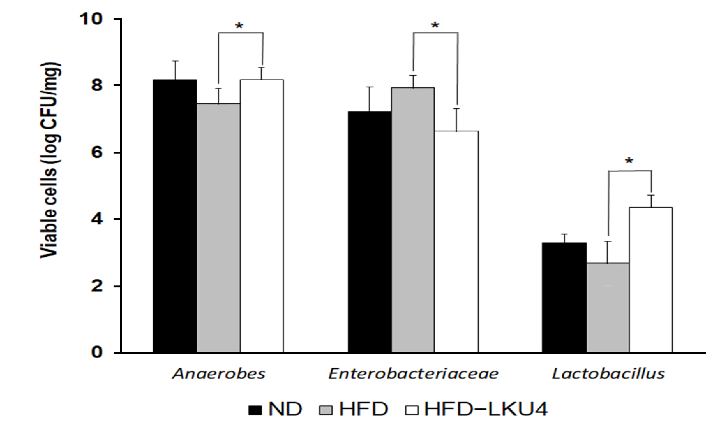


Supplementary Figure 2


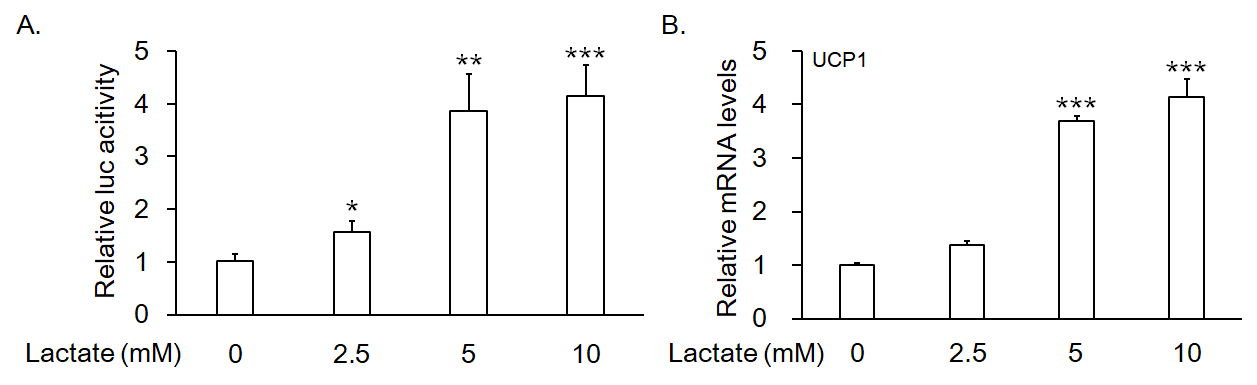


Supplementary Figure 3


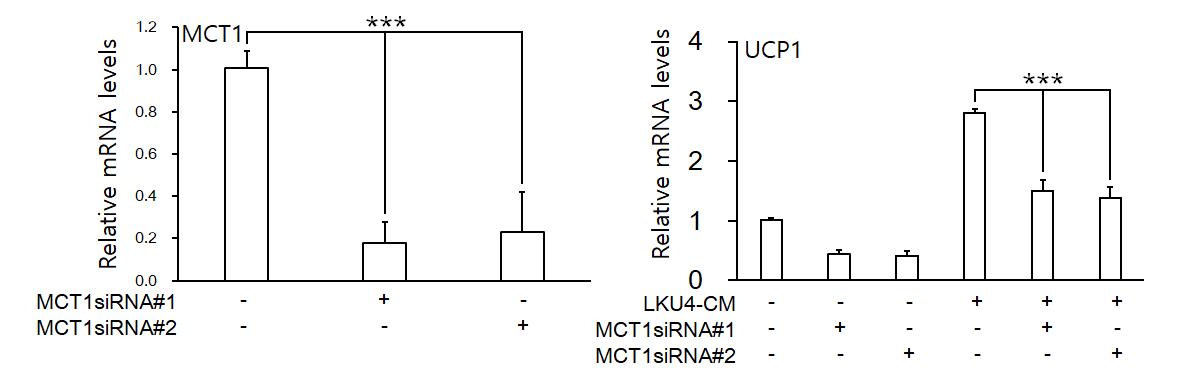


Supplementary Figure 4


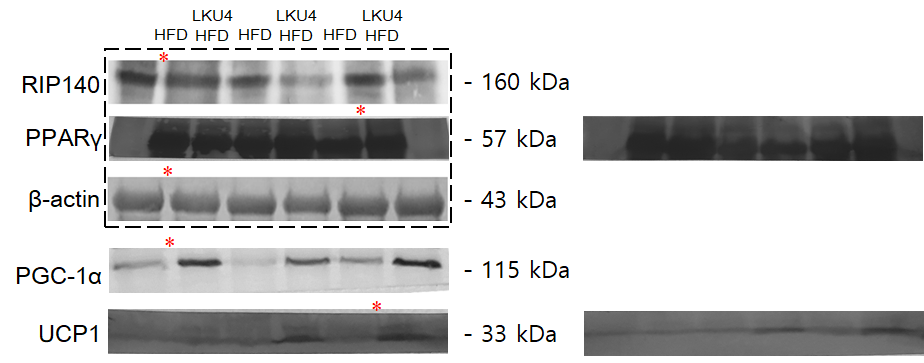


Supplementary Figure 5


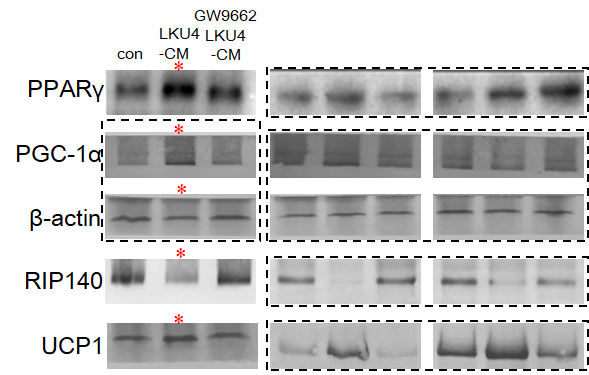


Supplementary Figure 6


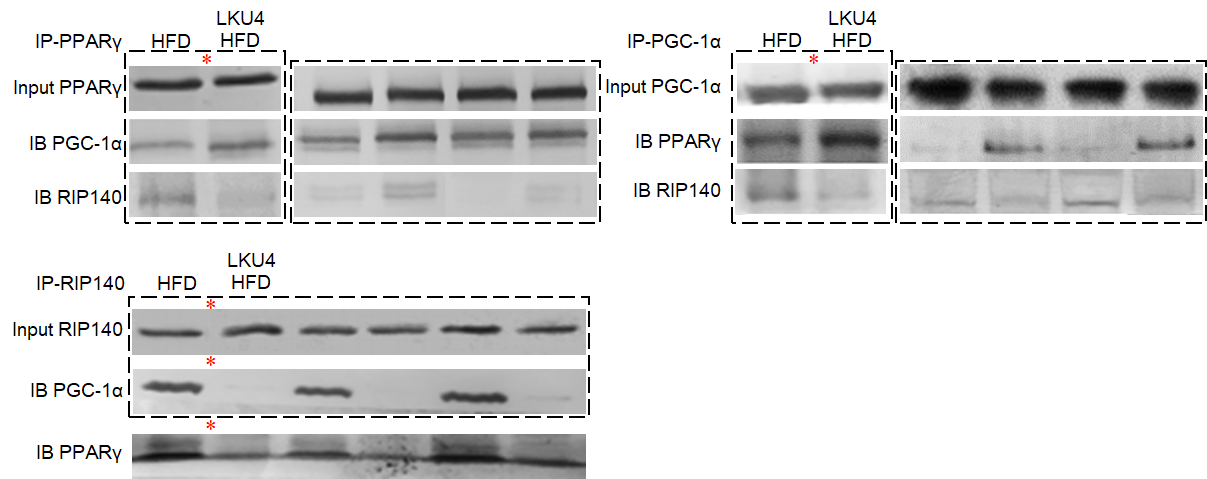


Supplementary Figure 7


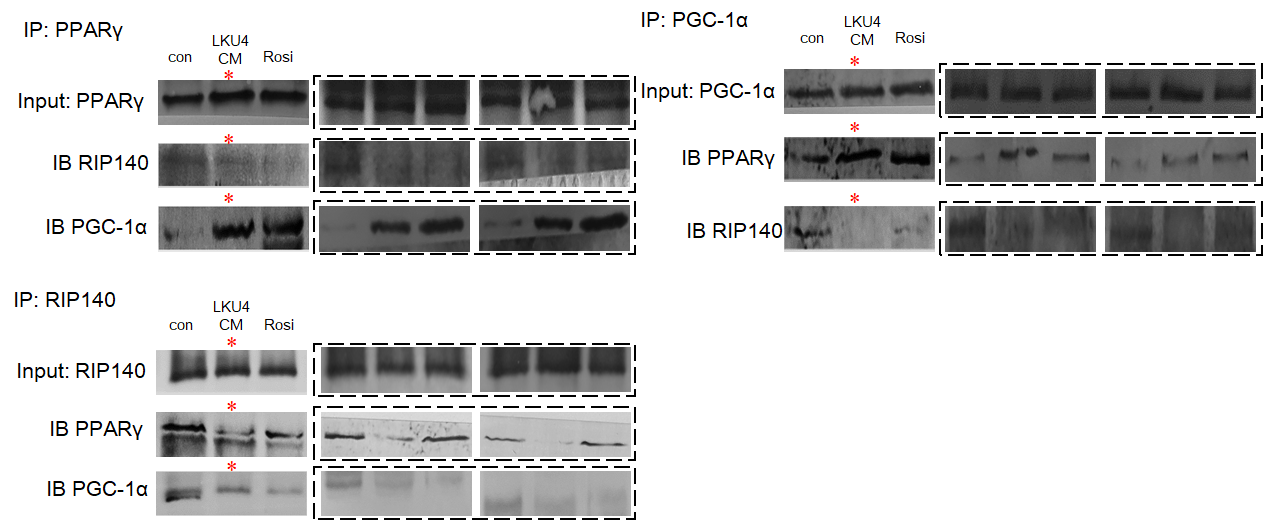


Supplementary Figure 8


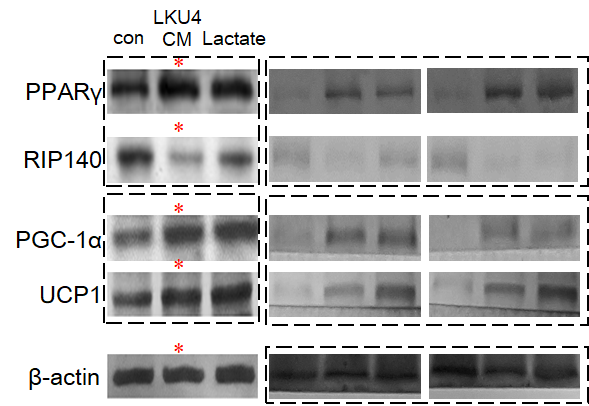


Supplementary Figure 9


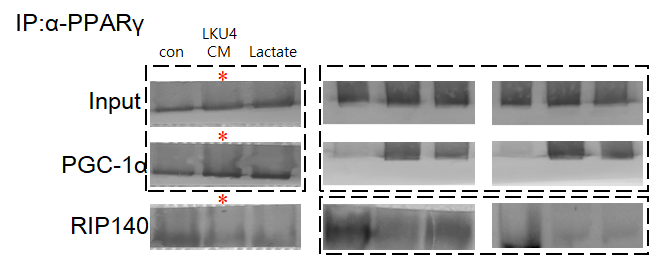


Supplementary **Table 1. Primers used for the RT-qPCR analysis**

| Gene | 5′-forward primer-3′ | 5′-reverse primer-3′ |
| --- | --- | --- |
| *PPARγ*  *PGC-1α*  *Prdm16*  *RIP140*  *Cidea*  *Ucp1*  *36B4* | TGCTGTTATGGGTGAAACTCTGGG  TCCTCTGACCCCAGACTCAC  CAGCACGGTGAAGCCATTC  GCTTGGCTCTGATGTGCATC  TAGCCAGAGTCACCTTCGAC  GGAGGTGTGGCAGTGTTC  AGATGCAGCAGATCCGCAT | CGCTTGATGTCAAAGGAATGCG  TAGAGTCTTGGAGCTCCT  GCGTGCATCCGCTTGTG  TCTTTGTGGCCAGCAGACTT  ATGGCTGCTCTTCTGTATCG  TCTGTGGTGGCTATAACTCTG  ATATGAGGCAGCAGTTTCTCCAG |
| mtDNA primer | 5′-forward primer-3′ | 5′-reverse primer-3′ |
|  | ACCATTTGCAGACGCCATAA | TGAAATTGTTTGGGCTACGG |
| ChIP primer | 5′- forward primer-3′ | 5′-reverse primer-3′ |
| PPRE  (*Ucp1* promoter) | GGGTGCCCTGTAAATGGTGT | GAGAGAGCAGTAGGGGTGAG |
